# Effect of Ionic Liquid on Silver-Nanoparticle-Complexed *Ganoderma applanatum* and Its Topical Film Formulation

**DOI:** 10.3390/pharmaceutics15041098

**Published:** 2023-03-29

**Authors:** Pattwat Maneewattanapinyo, Wiwat Pichayakorn, Chaowalit Monton, Nattakan Dangmanee, Thaniya Wunnakup, Jirapornchai Suksaeree

**Affiliations:** 1 Department of Pharmaceutical Chemistry, College of Pharmacy, Rangsit University, Muang 12000, Pathum Thani, Thailand; 2 Department of Pharmaceutical Technology, Faculty of Pharmaceutical Sciences, Prince of Songkla University, Hat-Yai 90112, Songkhla, Thailand; 3 Drug and Herbal Product Research and Development Center, College of Pharmacy, Rangsit University, Muang 12000, Pathum Thani, Thailand; 4 Cosmetic Technology and Dietary Supplement Products Program, Faculty of Agro and Bio Industry, Thaksin University, Ban Pa Phayom 93210, Phatthalung, Thailand

**Keywords:** ionic liquid, silver nanoparticles, *Ganoderma applanatum*, topical film

## Abstract

Imidazolium-based ionic liquids have been widely utilized as versatile solvents for metal nanoparticle preparation. Silver nanoparticles and *Ganoderma applanatum* have displayed potent antimicrobial activities. This work aimed to study the effect of 1-butyl-3-methylimidazolium bromide-based ionic liquid on the silver-nanoparticle-complexed *G. applanatum* and its topical film. The ratio and conditions for preparation were optimized by the design of the experiments. The optimal ratio was silver nanoparticles: *G. applanatum* extract: ionic liquid at 97:1:2, and the conditions were 80 °C for 1 h. The prediction was corrected with a low percentage error. The optimized formula was loaded into a topical film made of polyvinyl alcohol and Eudragit^®^, and its properties were evaluated. The topical film was uniform, smooth, and compact and had other desired characteristics. The topical film was able to control the release of silver-nanoparticle-complexed *G. applanatum* from the matrix layer. Higuchi’s model was used to fit the kinetic of the release. The skin permeability of the silver-nanoparticle-complexed *G. applanatum* was improved by about 1.7 times by the ionic liquid, which might increase solubility. The produced film is suitable for topical applications and may be utilized in the development of potential future therapeutic agents for the treatment of diseases.

## 1. Introduction

Because silver nanoparticles have many uses in different fields, such as biomedical sciences, drug delivery, and cosmetics, their synthesis is now very common. The optical and catalytic properties of silver nanoparticles, for example, depend on the size and shape of the particles produced. As a result, numerous researchers have noted the production of silver nanoparticles in various shapes that have numerous applications in areas such as medicine [1]. A few years ago, silver and its compounds were used in medicine as a bactericidal agent that is safe and non-toxic. A number of physical and chemical techniques for creating silver nanoparticles have been developed, but biological techniques, including employing plant and fungus material, are far simpler, less risky, and more environmentally benign [2]. The green synthesis of silver nanoparticles utilizing *Ganoderma applanatum* extract as a reducing and capping agent, also called “silver-nanoparticle-complexed *G. applanatum*”, followed by the structural and morphological characterization of the generated silver nanoparticles. *G. applanatum* has high antioxidant capacity and antimicrobial qualities, which include antibacterial and antifungal activities against pathogenic bacteria, which have led to its use in ethnomedicine [3,4,5]. Hence, silver-nanoparticle-complexed *G. applanatum* may demonstrate antibacterial and antifungal potentials.

One *Ganoderma* species is the widely distributed perennial bracket fungus known as *G. applanatum*. Traditional Asian medicines have primarily used the macrofungus genus *Ganoderma*, which belongs to the family *Ganodermataceae*, as a source of medicinal mushrooms rather than food to treat a variety of ailments. It has high genetic diversity [6]. *G. applanatum* is a higher-order medicinal basidiomycete mushroom that combats cancer in a variety of ways [7,8,9]. Microbial infection is a problem for both the global healthcare and agricultural sectors. In order to overcome this, it is essential to develop novel antimicrobial agents with a range of characteristics, such as antimicrobial potency, high compatibility, and low toxicity [10,11,12]. In this study, we synthesized silver nanoparticles using *G. applanatum* extract and an ionic liquid and characterized the generated silver nanoparticles. However, the obtained silver-nanoparticle-complexed *G. applanatum* has restricted permeability, making it difficult to develop effective topical and transdermal formulations for these substances. Ionic liquids have been interested in their possible use in pharmaceutics and medicine as a penetration enhancer because they have been reported to have biological properties.

Ionic liquids have long hydrophobic tails and typically exhibit activity similar to that of surfactants. Ionic liquids are capable of producing a wide range of molecular assemblies, including reverse micelles, regular micelles, and vesicles [13]. Through the fluidization of the lipid bilayer or the disruption of cellular structure, as well as through the creation of permeation routes, surface active ionic liquids have demonstrated great potential for facilitating drug transport across the skin or avoiding the physical barrier of the stratum corneum [14]. Numerous studies have been conducted to understand the underlying mechanisms of the action of ionic liquids because it has been demonstrated that they facilitate drug permeation through the skin [15,16,17,18]. The chemical composition of ionic liquids is implicated in several mechanisms. An ionic liquid’s level of permeation is particularly influenced by its structure. The transdermal permeation of the diltiazem-free base is significantly enhanced by treatment with all ionic liquids, and the levels of diltiazem hydrochloride in the receiving phase have been found to vary significantly depending on the ionic liquid structure. The best enhancer for both salt and free base drug forms is N-dodecyldabco bromide, despite it having some toxicity. The cytotoxicity and enhancer activity of N-methyl-N-decylmorpholinium bromide, in contrast, are well-balanced [19]. The traditional O/W and W/O emulsions with two ionic liquids, hydrophilic 1-hexyl-3-methylimidazolium chloride, and hydrophobic 1-butyl-3-methylimidazolium hexafluorophosphate components, have been created. There is a penetration improvement when ionic liquids are present. In particular, lipophilic substances have been estimated to have greater efficiency in penetrating into the deeper skin layers in comparison to hydrophobic 1-butyl-3-methylimidazolium hexafluorophosphate [20].

The silver-nanoparticle-complexed *G. applanatum* with ionic liquid as an active drug will thus be loaded in the topical film. A topical film is applied to a specific area of the body, usually the skin, to treat fungal infections by killing or stopping the growth of dangerous fungi on the skin. Topical drug delivery systems are external dosage forms that are administered to the skin. The physical states of topical dosage forms are then further divided into solid, liquid, and semisolid categories. Although topical drugs are meant to work locally, they can potentially have systemic effects [21].

In this study, silver nanoparticles and *G. applanatum* extracts were synthesized by the green technique to produce the nanoparticles. The 1-butyl-3-methylimidazolium bromide-based ionic liquid was incorporated into the silver-nanoparticle-complexed *G. applanatum*. The ratios of ingredients and the conditions for preparation were optimized by the design of the experiments. The particle size was a dependent variable for optimization. The characteristics of the optimized recipe of the silver-nanoparticle-complexed *G. applanatum* were investigated. The silver-nanoparticle-complexed *G. applanatum* was loaded into the topical film formulation. Folding endurance, Fourier-transform infrared spectroscopy (FTIR), a differential scanning calorimeter (DSC), thermogravimetric analysis (TGA), an X-ray diffractometer (XRD), a scanning electron microscope (SEM), and transmission electron microscopy (TEM) were all employed. The effects of the content of 1-butyl-3-methylimidazolium bromide on silver-nanoparticle-complexed *G. applanatum* release and permeation were assessed.

## 2. Materials and Methods

### 2.1. Preparation of G. applanatum Extract

**Figure 1:**
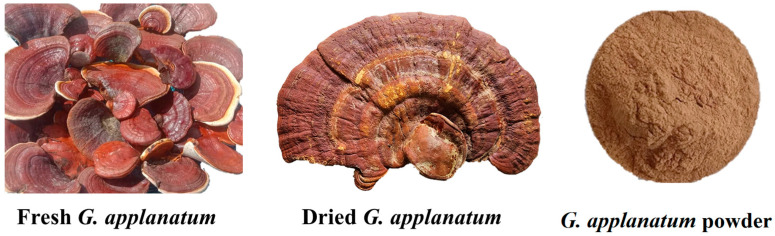
The appearance of *G. applanatum* in different forms.

*G. applanatum* (Figure 1) was taken in August 2022 in the forest region of Rattanaburi District, Surin Province, Thailand (15°18′11.7″ N 103°50′52.4″ E). Niran Vipunngern, taxonomist, Department of Pharmacognosy, College of Pharmacy, Rangsit University, identified the morphology of *G. applanatum*. The voucher specimen of *G. applanatum* was submitted to the Drug and Herbal Product Research and Development Center, College of Pharmacy, Rangsit University, and was given the identification number JS-GA1-08-2022. To avoid microbiological contamination, the fresh *G. applanatum* was carefully rinsed 2–3 times with flowing distilled water and then with 99.8% pure ethanol. The samples were immediately sliced into small pieces and dried in the shade at room temperature for six to seven days. An electric blender was used to obtain a fine-powdered form from dried materials.

In a beaker, 5 g of *G. applanatum* powder was extracted with 200 mL of methanol. After this, the extraction was carried out in a fume hood with a microwave oven (MS23F300EEK/ST model, triple distribution system, Samsung Electronics Co., Ltd., Selangor, Malaysia). To avoid overheating the extraction solvent, 60 s of intermittent microwave radiation (“on”) was followed by 60 s of non-heating (“off”). As a result, the total extraction time for each cycle was 120 s. In the four microwave-assisted extraction cycles, the microwave-assisted extraction power was 450 watts. The extraction solution had a temperature of 76 ± 2 °C, as measured with a glass laboratory thermometer. Following the separation process, the liquid phase was filtered through 0.45 μm Whatman No. 1 filter paper before being concentrated using a rotary evaporator at 40–60 °C under vacuum. The crude *G. applanatum* extracts were stored in a fume hood until their weights were constant and they were protected from light. The phytochemical screening found carbohydrates, glycosides, proteins, alkaloids, steroids, triterpenes, phenols, flavonoids, tannins, saponins, and lipids in *G. applanatum* extracts, findings which will be followed up on in a currently unpublished manuscript.

### 2.2. Biosynthesis of Silver-Nanoparticle-Complexed G. applanatum

Our approach for producing silver-nanoparticle-complexed *G. applanatum* was slightly modified from the prior method used [22]. Silver-nanoparticle-complexed *G. applanatum* was prepared by combining 15 mg/mL of *G. applanatum* extract and ionic liquid with 0.1 M silver nitrate (169.87 g/mol) solution in different mass ratios (as shown in Table 1), stabilizing the solution with starch soluble, and then stirring it on a hot plate at 80 °C with a magnetic stirrer for 2 h. The solution was then cooled to room temperature. The ratios of silver nanoparticles (*X*_1_), *G. applanatum* extract (*X*_2_), and ionic liquid (*X*_3_) were prepared, optimized, and predicted (Table 1) by the Design-Expert^®^ program (Stat-Ease, Inc., Minneapolis, MN, USA). The computer-generated linear model is presented as Equation (1). Then, the optimized ratio formula shown in Table 1 was used to optimize and predict the temperature (*X*_4_) and time (*X*_5_) for the preparation of silver-nanoparticle-complexed *G. applanatum* (Table 2). The computer-generated linear model is presented as Equation (2).

*Y_i_* = b_0_ + b_1_*X*_1_ + b_2_*X*_2_ + b_3_*X*_3_ (1)

*Y_i_* = b_0_ + b_4_*X*_4_ + b_5_*X*_5_ (2)

where *Y_i_* is the reasonable response (dependent variable) related to each factor level combined effect, b_0_ is an intercept, b_1_ to b_5_ are approximated regression coefficients calculated from the observed experimental values of *Y_i_*, and *X*_1_, *X*_2_, *X*_3_, *X*_4_, and *X*_5_ are the coded levels of the independent variables. The primary effects (*X*_1_, *X*_2_, *X*_3_, *X*_4_, and *X*_5_) reflect the typical outcomes of varying each factor from a low to a high level. The dependent variable (hydrodynamic particle diameter) and independent variables (silver nanoparticles, *G. applanatum* extract, ionic liquid, temperature, and time) were used for silver-nanoparticle-complexed *G. applanatum* preparation.

#### 2.2.1. Particle Size

The silver-nanoparticle-complexed *G. applanatum* was diluted ten times with ultrapure water before particle size measurement was carried out using NanoPlus-3 (Micromeritics, Particulate Systems, Norcross, GA, USA). The particle size of the sample was determined using dynamic light scattering. Each sample was measured three times.

#### 2.2.2. UV–Vis Spectra

A color change first indicated the presence of silver-nanoparticle-complexed *G. applanatum*. A UV/Visible scanning spectrophotometer (UV-1800, Shimadzu Corporation, Kyoto, Japan) was used at 420 nm with spectra ranging from 300 to 700 nm at room temperature, confirming the detection of *G. applanatum* by silver nanoparticles.

#### 2.2.3. Antioxidant Activity Assay

The scavenging activity of 2,2-diphenyl-1-picrylhydrazyl hydrate (DPPH) (%) was determined using a previously published method [23]. In a 96-well plate, 100 microliters of the sample and quercetin were prepared and diluted, 100 L of DPPH was added, and the mixture was incubated in a dark room for 30 min. After mixing for 10 s, the reduction in color intensity caused by free radicals was measured at 517 nm. Quercetin concentrations ranging from 0.031 to 0.50 mg/mL were used. A sample with concentrations ranging from 0.031 to 1.00 mg/mL was then prepared. Data from three replicates were used to calculate the mean.

The total antioxidant content of the sample was also determined using the ferric reducing antioxidant power (FRAP) assay. In a 96-well plate, 20 microliters of the sample and ferrous sulfate heptahydrate were prepared and diluted, 180 L of FRAP reagent was then added, and the mixture was incubated at 37 °C for 6 min. Sample absorbance was measured at 593 nm. Concentrations of ferrous sulfate heptahydrate ranging from 0 to 278 µM were employed. The sample was prepared at concentrations ranging from 0 to 1 mg/mL. Data for three replicates were used to calculate the mean total antioxidant content based on IC_50_.

#### 2.2.4. SEM and TEM

The sample was dropped on a copper stub and then kept at room temperature to evaporate the solvent. The sample was characterized by SEM, which was connected to an Everhart–Thornley detector (FESEM, Apreo, FEI, Amsterdam, The Netherlands).

The sample was fixed in 2.5% glutaraldehyde at room temperature overnight and then dehydrated in gradient alcohol (10–95%) for 20 min, followed by absolute alcohol for 2–5 min. The final specimen was coated with monolayer platinum to make the surface conduct. Field emission TEM was performed on a Thermo Scientific Talos F200i (FEI, Thermo Fisher Scientific, Waltham, MA, USA) operated at 200 kV accelerating voltage, and an AMT XR41-B 4-megapixel (2048 × 2048) CCD camera was used to image the sample.

### 2.3. Preparation of Topical Film Loaded with Silver-Nanoparticle-Complexed G. applanatum

A 10% *w*/*w* polyvinyl alcohol (PVA) solution was prepared in distilled water. PVA pellets (*Mw* 195,000, Sigma-Aldrich, St. Louis, MO, USA) were dissolved in hot distilled water and stirred continuously with a magnetic bar. The solution was allowed to cool to room temperature before being adjusted to 10% *w*/*w* with distilled water. Four grams of Eudragit^®^ NM 30D (Jebsen & Jessen NutriLife, Bangkok, Thailand) and two grams of glycerin (P.C. drug center, Bangkok, Thailand) were combined in twenty grams of 10% *w*/*w* PVA solution. Two grams of silver-nanoparticle-complexed *G. applanatum* were then slowly incorporated into the polymer solution. About 25 g of the solution was poured into a Petri dish and dried at 70 ± 2 °C in a hot air oven (model JSOF-100, Gongju-City, Korea) until a complete film was obtained.

#### 2.3.1. Characterization of Topical Film Loaded with Silver-Nanoparticle-Complexed *G. applanatum*

##### Folding Endurance

Folding the film sample repeatedly in the same area until it broke was used to test its folding endurance. The folding endurance was determined by counting the number of folds carried out before the film sample broke [24,25].

##### FTIR Spectrum

FTIR spectrometer (Vertex70, Bruker, Berlin, Germany) was used to examine the sample. The sample was scanned at a resolution of 4 cm^−1^ with 16 scans over a wavenumber region of 400–4000 cm^−1^. FTIR spectrum was recorded in absorption mode.

##### DSC

DSC equipment (DSC3+, METTLER TOLEDO, Greifensee, Switzerland) was used to examine the temperature behavior of the film sample at a rate of 10 °C/min from 25 °C to 400 °C. After being placed on an aluminum pan, the film sample was hermetically sealed. The DSC curves of the thermal properties of the film sample were obtained and reported.

##### TGA

TGA was performed in a TGA/DSC3+ (METTLER TOLEDO, Greifensee, Switzerland) from 50 to 650 °C under a nitrogen atmosphere (100 mL/min). Samples (5–10 mg) were analyzed in a TGA pan at a heating rate of 10 °C/min. TGA thermograms were produced, and the DTG was calculated.

##### XRD

An XRD instrument (Empyrean, PANalytical, Almelo, The Netherlands) was used to examine the crystallinity of the film sample. The operating voltage of the generator was 40 kV, and the current of the X-ray source was 45 mA with a stepped angle of 0.02°/s in the angular range of 5–40°.

##### SEM

An SEM connected to an Everhart–Thornley detector (FESEM, Apreo, FEI, Eindhoven, The Netherlands) was used to examine the cross-sectional morphology of the film sample. The produced film sample was directly coated with gold after being put on a copper stub.

#### 2.3.2. Determination of the Content of Silver-Nanoparticle-Complexed *G. applanatum* in Topical Film

A total of 1 cm × 1 cm was taken from five different locations of the topical film loaded with silver-nanoparticle-complexed *G. applanatum*, which divided the film into small pieces. The sample was soaked in distilled water for 30 min before being sonicated. The solution was collected and diluted with distilled water to the appropriate concentration before being measured with a UV/Visible scanning spectrophotometer (UV-1800, Shimadzu Corporation, Kyoto, Japan) at 420 nm with spectra ranging from 300 to 700 nm at room temperature. The drug content average and standard deviation were reported.

#### 2.3.3. In Vitro Release of Silver-Nanoparticle-Complexed *G. applanatum* from Topical Film

A total of 2 cm × 2 cm of topical film loaded with silver-nanoparticle-complexed *G. applanatum* was applied on the donor compartment of vertical Franz diffusion cells (EMFDC06, Orchid Scientific, Maharashtra, India) with an effective diffusion area of 1.77 cm^2^. The Franz cells were equipped with a dialysis cellulose membrane (MWCO: 3500 Da, CelluSep^®^ T4, Membrane Filtration Product, Inc., Seguin, TX, USA), which was soaked overnight in a receptor medium at 32 ± 0.5 °C before use. The receptor medium was 12 mL of isotonic phosphate-buffered solution at pH 7.4 with a water jacket at 37 ± 0.5 °C, which was stirred constantly at 600 rpm with a magnetic stirrer. One mL of the receptor medium was withdrawn at 0.5, 1, 2, 4, 6, 8, 10, 12, and 24 h, and then this was immediately replaced by an equal volume of fresh isotonic phosphate-buffered solution at pH 7.4. The content of silver-nanoparticle-complexed G. applanatum was analyzed by the UV/Visible scanning spectrophotometer (UV-1800, Shimadzu Corporation, Kyoto, Japan), which was operated at 420 nm using spectra ranging from 300 to 700 nm at room temperature. The experiments for each sample were performed in triplicate.

#### 2.3.4. Kinetic Models

Different mathematical models, namely the zero-order, first-order, and Higuchi empirical models, and the semi-empirical Korsmeyer–Peppas model, were used to characterize the silver-nanoparticle-complexed *G. applanatum* release kinetics. DDSolver was used to assess the kinetic models based on four models [26] following Equations (3)–(6).

(3) $$\mathrm{Zero}\text{-}\mathrm{order}\;\mathrm{model}\;Q_{t}=Q_{0}+\;K_{0}t$$

(4) $$\mathrm{First}\text{-}\mathrm{order}\;\mathrm{model}\;\ln\;Q_{t}=\ln\;Q_{0}-K_{1}t$$

(5) $$\mathrm{Higuchi}’s\;\mathrm{model}\;Q_{t}=K_{H}\sqrt{t}$$

(6) $$\mathrm{Korsmeyer}\text{–}\mathrm{Peppas}\;\mathrm{model}\;\frac{Q_{t}}{Q_{0}}=K_{\mathrm{KP}}t^{n}$$

where *Q*_0_ was the amount of initial drug and *Q_t_* was the amount of drug release or permeation in time (*t*).

#### 2.3.5. In Vitro Permeation of Silver-Nanoparticle-Complexed *G. applanatum* from Topical Film

The 2 cm × 2 cm sample of topical film loaded with silver-nanoparticle-complexed G. applanatum was applied on the donor compartment of the vertical Franz diffusion cells (EMFDC06, Orchid Scientific, India) with an effective diffusion area of 1.77 cm^2^. The Franz cells were equipped with a dead pig ear skin sample that varied from 300 to 450 μm in thickness, which was soaked overnight in a receptor medium at 32 ± 0.5 °C before use. The receptor medium was 12 mL of isotonic phosphate-buffered solution at pH 7.4 with a water jacket at 37 ± 0.5 °C, which was stirred constantly at 600 rpm with a magnetic stirrer. One mL of the receptor medium was withdrawn at 0.5, 1, 2, 4, 6, 8, 10, 12, and 24 h, with this immediately being replaced by an equal volume of fresh isotonic phosphate-buffered solution at pH 7.4. The content of silver-nanoparticle-complexed G. applanatum was analyzed by the UV/Visible scanning spectrophotometer (UV-1800, Shimadzu Corporation, Kyoto, Japan), which was operated at 420 nm using spectra ranging from 300 to 700 nm at room temperature. The experiments for each sample were performed in triplicate.

The dead pig ear skin samples were taken out of the diffusion cells at the end of the in vitro skin permeation experiment and washed twice with 1 mL of distilled water to remove any residual residue from the skin’s surface. Every skin sample was divided into small pieces before being extracted in distilled water by homogenization and being sonicated for 60 min. Samples were centrifuged, and the supernatant was collected and then filtered through 0.45 µm syringe filters; the samples were then analyzed with the UV/Visible scanning spectrophotometer.

## 3. Results and Discussion

### 3.1. Optimization of Composition and Preparation Conditions of Silver-Nanoparticle-Complexed G. applanatum

**Figure 2:**
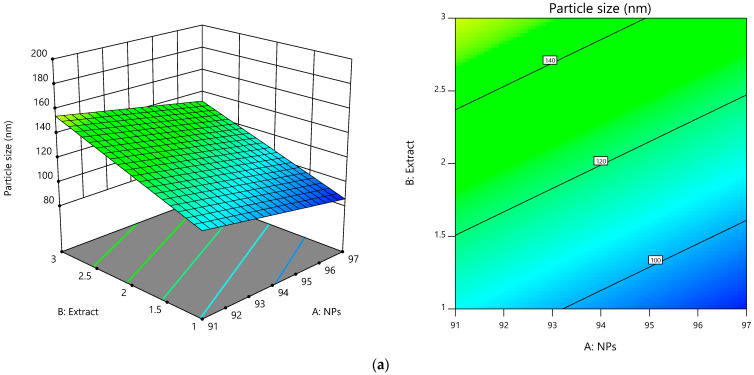

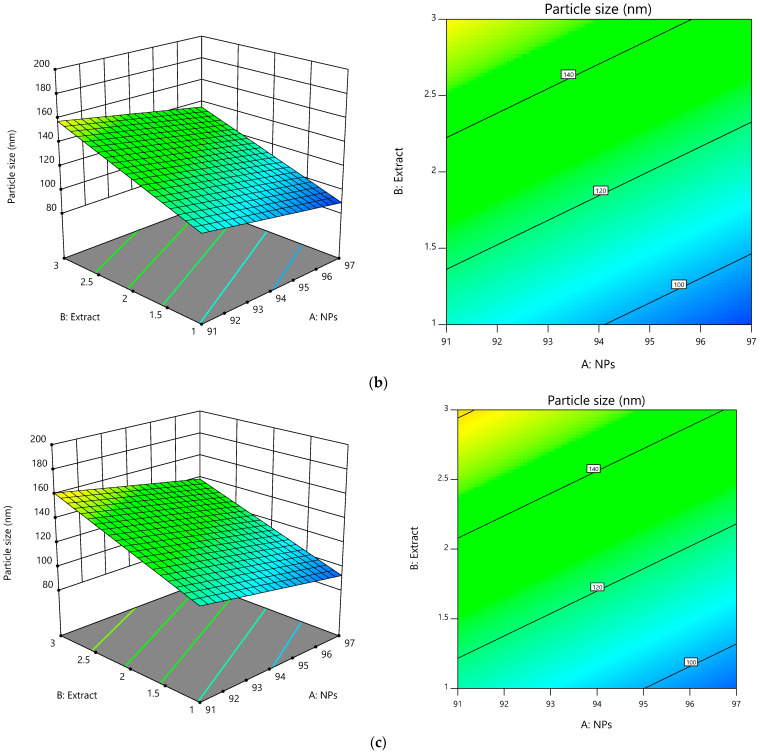
(left) 3D response surface and (right) contour plot of model conditions of silver-nanoparticle-complexed *G. applanatum*: silver nanoparticles (*X*_1_), *G. applanatum* extract (*X*_2_), and ionic liquid (*X*_3_) as independent variables, and particle size as a dependent variable. (**a**) *X*_3_ = 2, (**b**) *X*_3_ = 4, and (**c**) *X*_3_ = 6.

**Figure 3:**
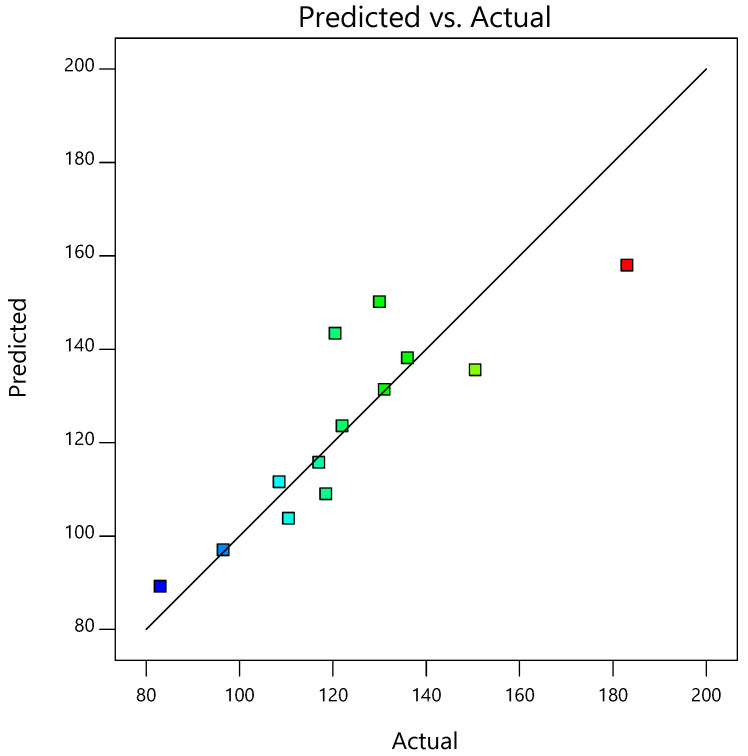
Predicted versus actual plots of model conditions of silver-nanoparticle-complexed *G. applanatum*: silver nanoparticles (*X*_1_), *G. applanatum* extract (*X*_2_), and ionic liquid (*X*_3_) as independent variables, and particle size as a dependent variable.

For the preparation of the silver-nanoparticle-complexed *G. applanatum*, the particle size was optimized by the Design-Expert^®^ program. Figure 2 shows the predicted ratios of the silver nanoparticles, *G. applanatum* extract, and ionic liquid in different combinations (Table 1). The linearity plot of the model ratios of the components’ predicted values vs. actual values is shown in Figure 3, which indicates a great correlation between the linear model and the data. The actual equation used to predict each dependent variable was Y = 421.03 − 3.73 (silver nanoparticles) + 23.19 (*G. applanatum* extract) + 1.69 (ionic liquid). The optimal ratio of the silver nanoparticles, *G. applanatum* extract, and the ionic liquid were 97:1:2. The ANOVA findings, which had an adequate precision of 8.3733, confirmed the model’s significance. Both the “predicted R-squared” value of 0.9994 and the “adjusted R-squared” value of 0.9430 were quite accurate. The probability of obtaining a significant “model F-value” due to noise was 0.01%, and the model’s F-value of 8.21 indicated that the model was significant. Furthermore, the signal-to-noise ratio was measured, and the range of the predicted values at the design points was compared to the average prediction error (a ratio larger than four is regarded as acceptable) [27]. As this ratio was found to be greater than four, this demonstrated that the model was suitable for predicting the findings inside the design space without the need for additional trials. The prediction value of the particle size for this optimal ratio was 85.87 nm. Next, the optimal ratio was then produced once again under the usage conditions of preparing the sample at 80 °C and agitating it for 2 h using a magnetic stirrer. This repeated preparation produced particles with a size of 91.50 ± 7.78 nm. [(Experimental value-predicted value/experimental value) × 100] was used to calculate the percent error of the prediction, which was found to be 6.15%.

**Figure 4:**
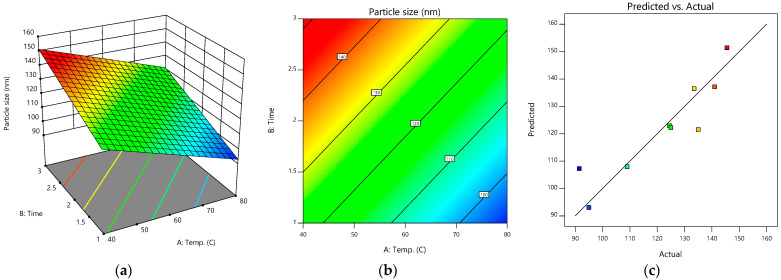
(**a**) 3D response surface, (**b**) contour plot, and (**c**) predicted versus actual plots of model conditions of silver-nanoparticle-complexed *G. applanatum*: temperature (*X*_4_) and time (*X*_5_) as independent variables and particle size as a dependent variable.

After the optimal ratio of the silver nanoparticles, *G. applanatum* extract, and ionic liquid at 97:1:2 was accepted, the preparation conditions were next optimized and forecasted by the Design-Expert^®^ program (Table 2). Figure 4a,b show the 3D response surface and contour plot of the predicted preparation conditions for the silver-nanoparticle-complexed *G. applanatum*. Figure 4c shows the linearity plot of the model for the preparation conditions as predicted values versus actual values, which showed a good correlation between the linear model and the data. The formula of Y = 138.47 − 0.75 (Temperature) − 14.25 (Time) was utilized to forecast each dependent variable. The ANOVA findings, which had an adequate precision of 11.0224, verified the significance of the model. The “predicted R-squared” value of 0.9896 and the “adjusted R-squared” value of 0.9802 were both extremely precise. The model’s F-value of 15.19 indicated that the model was significant, and the probability of a significant “model F-value” being obtained due to noise was 0.01%. The model was suitable for predicting the results inside the design space without the need for further trials, as shown by the fact that the ratio, in this case, was higher than four. The optimal preparation conditions for the silver-nanoparticle-complexed *G. applanatum* involved stirring the mixture at 80 °C for 1 h. Under these optimal preparation conditions, the predicted particle size was 93.06 nm. The optimal ratio for the silver-nanoparticle-complexed *G. applanatum* was then created again under the same conditions, which included preparing the mixture at 80 °C and stirring it for an hour with a magnetic stirrer. Particles measuring 97.50 ± 9.19 nm were obtained from this repeated preparation. The percent error of the prediction, which was found to be 4.55%, was calculated as [(Experimental value-predicted value/experimental value) × 100].

In summary, the Design-Expert^®^ program’s optimization of the composition and preparation conditions of the silver nanoparticles was successful in demonstrating the low percent error of the prediction—less than 10%—which was satisfactory; this optimization was therefore accepted and used for the subsequent experiments. The 97:1:2 ratio of the silver nanoparticles: *G. applanatum* extract: ionic liquid was prepared by heating the mixture to 80 °C and stirring it for an hour with a magnetic stirrer to produce the silver-nanoparticle-complexed *G. applanatum*, which was utilized in the subsequent experiments. The synthesized silver-nanoparticle-complexed *G. applanatum* was observed the color changes and its characterizations were evaluated.

**Figure 5:**
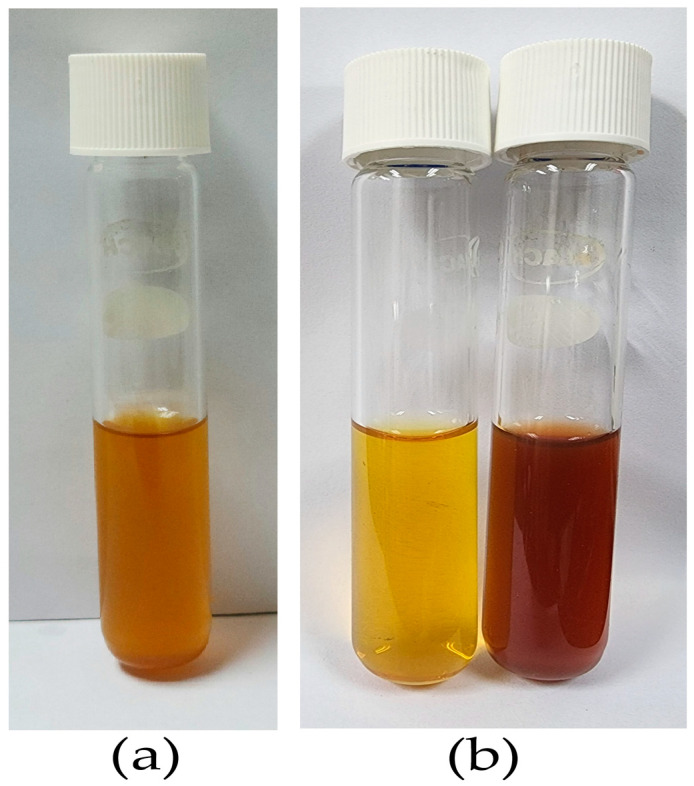
(**a**) Solution of pure *G. applanatum* extract and (**b**) silver nanoparticles were synthesized under optimal conditions: (left) without *G. applanatum* extract and (right) with *G. applanatum* extract.

To effectively develop silver nanoparticles, capping and reducing agents, as well as reaction conditions, are required. On the other hand, the significant variations in the biochemical components of each *G. applanatum* fungal species may have a massive influence on the silver nanoparticle biosynthesis procedure. The availability of produce enzymes that catalyze the reducing and capping processes of silver nanoparticles is thought to make eukaryotic organisms, such as plants, fungi, protists, and algae, suitable candidates for the “green synthesis” of silver nanoparticles [6,28,29,30]. Consequently, the current work focused on the “green synthesis” of silver nanoparticles utilizing *G. applanatum* extract as a reducing and capping agent, followed by the structural and morphological characterization of the generated silver nanoparticles. The silver nanoparticles were prepared again under the optimized conditions: the ratio between the silver nanoparticles: *G. applanatum* extract: ionic liquid was 97:1:2, and the mixture was stirred on a hot plate at 80 °C with a magnetic stirrer for 2 h. The varied colors of the prepared solution are shown in Figure 5. The solution of pure *G. applanatum* extract was light orange color (Figure 5a). Silver nitrate, which was utilized as the starting solution in the preparation, was clear and colorless, but the silver nanoparticles using 0.07 M of NaBH_4_ as a reducing agent were a light yellow color (Figure 5b, left). The color changes, which indicated the creation of these nanoparticles, were due to the surface plasmon resonance of the silver nanoparticles [30]. The silver-nanoparticle-complexed *G. applanatum*, which was clear and dark brown (Figure 5b, right), was prepared using *G. applanatum* extract in place of the 0.07 M of NaBH_4_.

#### 3.1.1. UV–Vis Spectra

**Figure 6:**
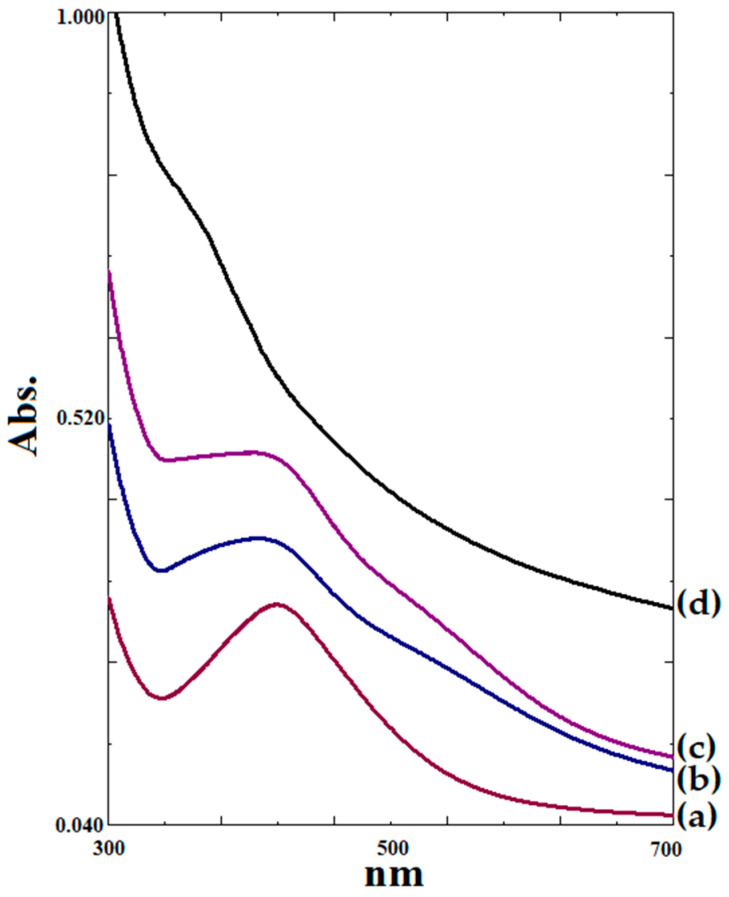
UV–Vis spectra of (a) silver nanoparticles without *G. applanatum* extract synthesized under optimal conditions, (b) silver-nanoparticle-complexed *G. applanatum* synthesized under optimal conditions, (c) silver-nanoparticle-complexed *G. applanatum* synthesized under optimal conditions after storage at room temperature for 1 month, and (d) *G. applanatum* extract.

The formation of silver-nanoparticle-complexed *G. applanatum* was observed using UV–visible spectroscopy. In previous studies, this approach has worked well for identifying surface plasmon resonance peaks. When the Ag electron is in the transmission band, it begins to vibrate in resonance with a specific wavelength of the light source, resulting in surface plasmon resonance peaks in silver nanoparticles [31]. The UV–visible absorption spectra of nanoparticles are shown in Figure 6, with the surface plasmon resonance peaking (maximum peak) at 419.5 nm for the silver nanoparticles without *G. applanatum* extract (Figure 6a) and 405.5 nm for the silver-nanoparticle-complexed *G. applanatum* (Figure 6b). The *G. applanatum* extract had high unexplained absorption in this UV–visible wavelength, suggesting that the extract might include several compounds (Figure 6d). Generally, silver nanoparticles have been found to have a significant and wide surface plasmon resonance peak between 410 and 450 nm, which was previously thought to be caused by spherical nanoparticles [10,32,33]. The primary absorption of the silver nanoparticle band was displaced to a lower absorption wavelength when the silver-nanoparticle-complexed *G. applanatum* was created, demonstrating that the synthesized silver-nanoparticle-complexed *G. applanatum* was an agglomeration and might have had various sizes and shapes. Furthermore, asymmetry and broadness were visible in the peak absorption of the silver-nanoparticle-complexed *G. applanatum*. This could have been affected by the incubation period and higher temperature, which have been found in a previous study to substantially increase the creation of silver-nanoparticle-complexed *G. applanatum* [6]. Additionally, the extracts may have led to an increase in agglomeration. The asymmetry and broadness of the peak absorption increased when the silver-nanoparticle-complexed *G. applanatum* was stored at room temperature for 1 month, which might have enhanced the agglomeration and size of the particles. However, it was observed that the maximum absorption at 405.5 nm did not change the unique absorption of the silver-nanoparticle-complexed *G. applanatum* (Figure 6c). The equation for the calibration curve for the silver-nanoparticle-complexed *G. applanatum* at 2.5–80 µg/mL was y = 0.0112x + 0.0282, and the linear regression coefficient (R^2^) was 0.9984. For this research work, this calibration curve was employed.

#### 3.1.2. Antioxidant Activity Assay

The IC_50_ antioxidant activity of the selected silver-nanoparticle-complexed *G. applanatum* determined from the DPPH assay was 419 ± 0.016 µg/mL, whereas the IC_50_ of quercetin (positive control) was 141 ± 0.004 µg/mL. The IC_50_ antioxidant activity of the selected silver-nanoparticle-complexed *G. applanatum* determined from the FRAP assay was 500 ± 0.04 µg/mL, whereas the IC_50_ of quercetin (positive control) was 88.12 ± 13.5 µg/mL.

#### 3.1.3. SEM

**Figure 7:**
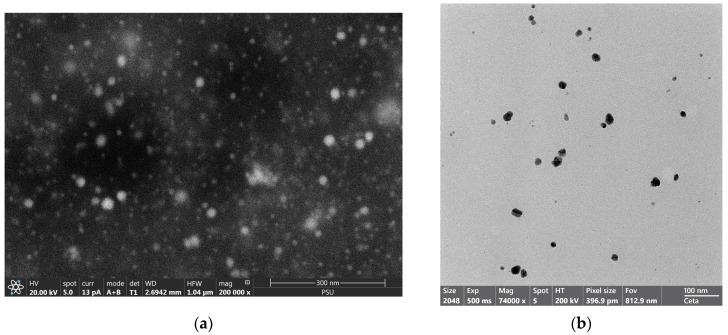
(**a**) SEM: 200,000× and (**b**) TEM: 74,000× images of silver-nanoparticle-complexed *G. applanatum* produced under optimal conditions.

SEM and TEM images of the silver-nanoparticle-complexed *G. applanatum* that was produced under optimal conditions are shown in Figure 7. It was found that the particles of the silver-nanoparticle-complexed *G. applanatum* formed in spherical nanoparticles with a size of 18.64 ± 5.32 nm that were distributed on the stub (Figure 7a). Further information on the shape of the silver-nanoparticle-complexed *G. applanatum* was also presented by TEM photograph. The TEM investigation of the silver-nanoparticle-complexed *G. applanatum*, which had an average diameter of 21.61 ± 6.53 nm, also confirmed their spherical shape (Figure 7b). From the above section, the particle size of the silver-nanoparticle-complexed *G. applanatum* that was produced under optimal conditions was 97.50 ± 9.19 nm. Because the effect of a hydrodynamic diameter could be seen in the particle, which was measured by dynamic light scattering using the NanoPlus-3 instrument. Since this layer affects the particle’s movement, the so-called hydrodynamic particle diameter is often larger than that measured, for instance, using SEM and TEM. The liquid’s electrical conductivity is one of several variables that affect the layer’s thickness. The hydrodynamic particle diameter of the primary particles or agglomerates in the liquid is critical for determining the behavior of nanoelements in a fluid [34]. Thus, its particle size could be bigger than that determined by other methods such as SEM and TEM. Moreover, the XRD crystalline size of the silver-nanoparticle-complexed *G. applanatum* was supported by both the SEM and TEM data.

### 3.2. Preparation of Topical Film Loaded with Silver-Nanoparticle-Complexed G. applanatum

After the silver-nanoparticle-complexed G. applanatum was successfully prepared and investigated under the abovementioned optimal conditions, it was loaded in a topical film consisting of PVA, Eudragit^®^ NM 30D as a polymer matrix, and glycerin as a plasticizer. The characterizations of a topical film were followed.

#### 3.2.1. Characterization of Topical Film Loaded with Silver-Nanoparticle-Complexed *G. applanatum*

##### Folding Endurance

**Figure 8:**
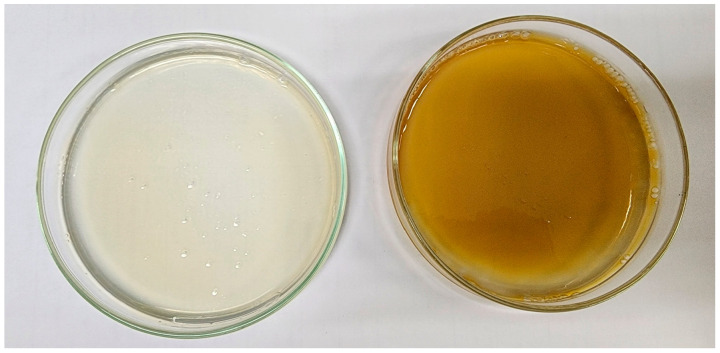
(**left**) Blank film and (**right**) topical film containing silver-nanoparticle-complexed *G. applanatum*.

The blank film was a smooth, translucent, colorless yellow (Figure 8, left). The loading of the silver-nanoparticle-complexed *G. applanatum* caused the topical film to become more yellow (Figure 8, right). This occurred due to the silver-nanoparticle-complexed *G. applanatum*’s characteristic color (Figure 5, right). The test for the folding endurance of film involves repeatedly folding a piece of the film until it breaks, demonstrating that the film will remain intact when applied to the skin [35]. In this study, the blank film and topical film were found to have folding endurance values of 716 ± 26 and 712 ± 24 folds, respectively. The addition of silver-nanoparticle-complexed *G. applanatum* is no significant effect on the folding endurance. However, the produced topical film was able to adhere effectively to the skin throughout all periods of usage.

##### FTIR Spectrum

**Figure 9:**
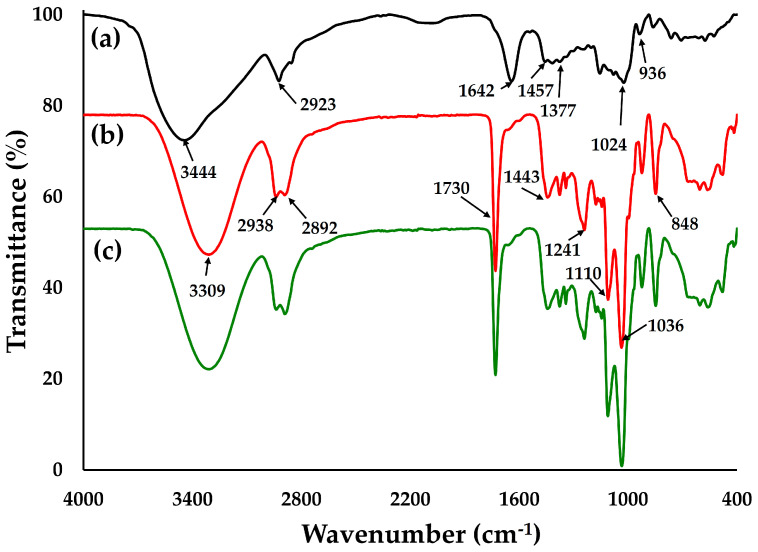
FTIR spectra of (a) silver-nanoparticle-complexed *G. applanatum*, (b) blank film, and (c) topical film containing silver-nanoparticle-complexed *G. applanatum*.

FTIR is a helpful technique for investigating the core–shell morphology of produced silver-nanoparticle-complexed *G. applanatum*. The method also extensively shows the chemical bonds and molecular structures involved in the stabilization and capping of silver-nanoparticle-complexed *G. applanatum*, as well as Ag^+^ bio-reduction [6]. The presence of a capping agent alongside the nanoparticles was found by the FTIR spectra shown in Figure 9a, which showed absorption bands at various wavenumbers. Table 3 provides a summary of the peak identification of the silver-nanoparticle-complexed *G. applanatum*. The presence of a capping agent alongside the nanoparticles was demonstrated by the absorption bands in the FTIR spectra at 3444, 2923, 1642, 1377, and 1024 cm^−1^. The main transmittance peaks of *G. applanatum* might have been located at 1024 cm^−1^. The primary absorption peaks of the blank film, which contained PVA, Eudragit^®^ NM 30D, and glycerin as ingredients, are shown in Figure 9b and Table 3. The primary absorption peaks of the topical film loaded with silver-nanoparticle-complexed *G. applanatum* (Figure 9c) were similar to those of the blank film (Figure 9b). This occurred because there was a smaller quantity of silver-nanoparticle-complexed *G. applanatum* than blank film; consequently, the topical film showed characteristics more similar to the blank film. However, a wider absorption was found at 3309 cm^−1^ for the topical film. This might have been the consequence of combining the characteristics of the silver-nanoparticle-complexed *G. applanatum* and the blank film.

##### DSC

**Figure 10:**
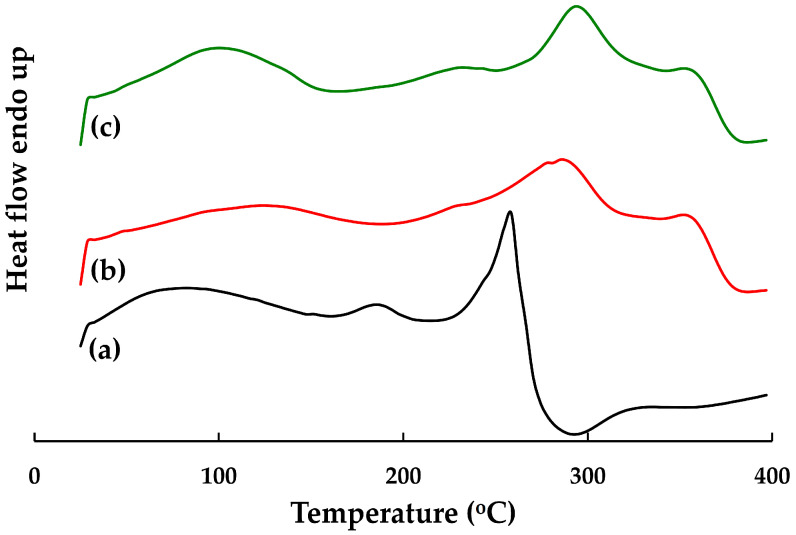
DSC thermograms of (a) silver-nanoparticle-complexed *G. applanatum*, (b) blank film, and (c) topical film containing silver-nanoparticle-complexed *G. applanatum*.

The main endothermic peaks of the silver-nanoparticle-complexed *G. applanatum*, blank film, and topical film were seen at 257.17, 286.50, and 294.17 °C, respectively, which can be interpreted as melting points (Figure 10). The minor endothermic peak of the topical film, which was seen at 229.50 °C, was broadened and shifted to lower temperatures in its curves, which was consistent with the presence of the silver-nanoparticle-complexed *G. applanatum*. Furthermore, there were no interactions or changes in the chemical structure of the topical film seen in the FTIR spectrum (Figure 9). Therefore, the silver-nanoparticle-complexed *G. applanatum* was often effectively entrapped in the topical film, with only minor shifts occurring in terms of widening or shifting towards a lower temperature of melting endothermic. These minor changes may just have been the result of the components being mixed, which reduces their purity but does not necessarily indicate a potential incompatibility.

##### TGA

**Figure 11:**
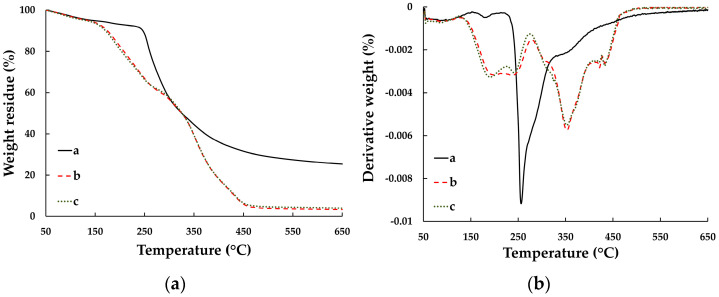
(**a**) TGA and (**b**) DTG thermograms of silver-nanoparticle-complexed *G. applanatum*, blank film, and topical film containing silver-nanoparticle-complexed *G. applanatum*.

The major weight losses of the silver-nanoparticle-complexed *G. applanatum*, blank film, and topical film were seen at 275.17, 335.17, and 336.89 °C, respectively (Figure 11), which may have been correlated with the DSC results. The topical film showed weight loss in the silver-nanoparticle-complexed *G. applanatum* at 250.73 °C. Therefore, this may have corroborated the presence of silver-nanoparticle-complexed *G. applanatum* in the topical film. Even though this weight loss was just a slight change, it had no effect because all the components of the topical film were compatible.

##### XRD

**Figure 12:**
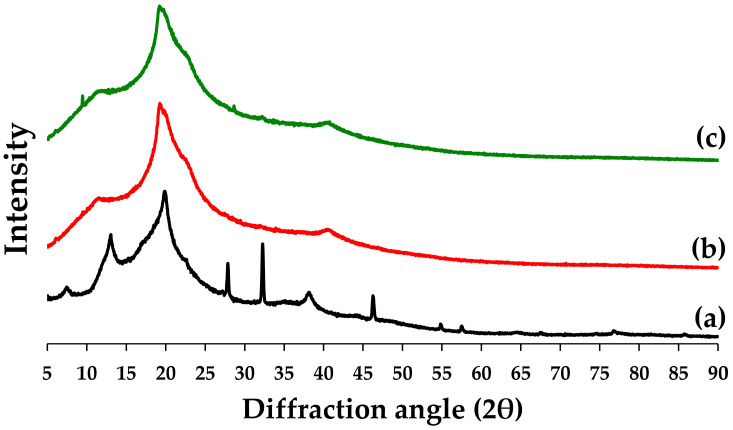
XRD patterns of (a) silver-nanoparticle-complexed *G. applanatum*, (b) blank film, and (c) topical film containing silver-nanoparticle-complexed *G. applanatum*.

Silver nanoparticles have been found in the literature to have crystal characteristics at 38.23°, 44.42°, 64.44°, and 77.37° (2θ), which correspond to the fcc planes (111), (200), (220), and (311), respectively, [10,36], whereas in this study, the crystal characteristics of the silver-nanoparticle-complexed *G. applanatum* were found at 7.55°, 13.02°, 19.85°, 27.87°, 32.27°, 38.17°, 44.24°, 46.23°, 64.46°, and 76.73° (2θ). The silver-nanoparticle-complexed *G. applanatum* displayed the distinctive crystal properties of silver nanoparticles (Figure 12a). Thus, the silver nanoparticles’ composition in the silver-nanoparticle-complexed *G. applanatum* was verified. PVA, the main polymer in the composition of the topical film, which was detected at 2θ = 19.15°, was apparent in the blank film through its main semi-crystalline characteristics (Figure 12b). Strong intramolecular and intermolecular hydrogen connections exist between PVA and the main chain because of the OH groups’ binding to the major chain [24,25]. In this study, because the silver-nanoparticle-complexed *G. applanatum* was present in significantly lower amounts than the blank film in the topical film, the topical film showed a semi-crystalline structure that was more closely related to the characteristics of the blank film (Figure 12c).

##### SEM

**Figure 13:**
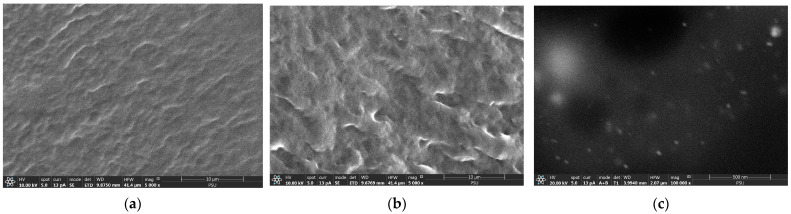
Cross-sectional morphologies of the blank film (**a**) at 5000× and topical film containing silver-nanoparticle-complexed *G. applanatum* (**b**) at 5000× and (**c**) 100,000×.

The cross-sectional morphologies of the blank film and topical film (magnified by 5000×) are shown in Figure 10b and Figure 13a, respectively. The image shown in Figure 13c, which was magnified by 100,000×, portrays the cross-sectional morphology of the topical film. The blank film had a smooth, compact layer that was absent of porosity, cracks, and cavities (Figure 13a). The layer of the topical film was denser than that of the blank film and had a roughness devoid of pores, cracks, and cavities (Figure 13b). However, the cross-sectional morphology of the topical film did not show the particles of the silver-nanoparticle-complexed *G. applanatum*; therefore, it was magnified at a factor of 100,000× (Figure 13c). The particles of the silver-nanoparticle-complexed *G. applanatum* were found to have small particles that were distributed throughout the topical film. Therefore, it can be assumed that the topical film had silver particles within it.

#### 3.2.2. Determination of the Content of Silver-Nanoparticle-Complexed *G. applanatum* in Topical Film

The topical film was produced with a silver-nanoparticle-complexed *G. applanatum* concentration of 49.09 ± 9.25 µg/cm^2^. In a previous study, a nanocomposite film with a surface area of 15 cm^2^ was found to contain 540 µg of silver content, had good mechanical strength and stability under storage for easy handling in an aqueous medium, and exhibited high trace metal sorption activity when subjected to optimum chemical conditions [37]. Therefore, in this study, the quantity of silver-nanoparticle-complexed *G. applanatum* in the topical film, which had a surface area of 15 cm^2^, was estimated to be 736.31 ± 138.82 µg, meaning it could be effectively used in applications. Additionally, this quantity in the topical film was more potent than the IC_50_ estimate.

#### 3.2.3. In Vitro Release of Silver-Nanoparticle-Complexed *G. applanatum* from Topical Film

**Figure 14:**
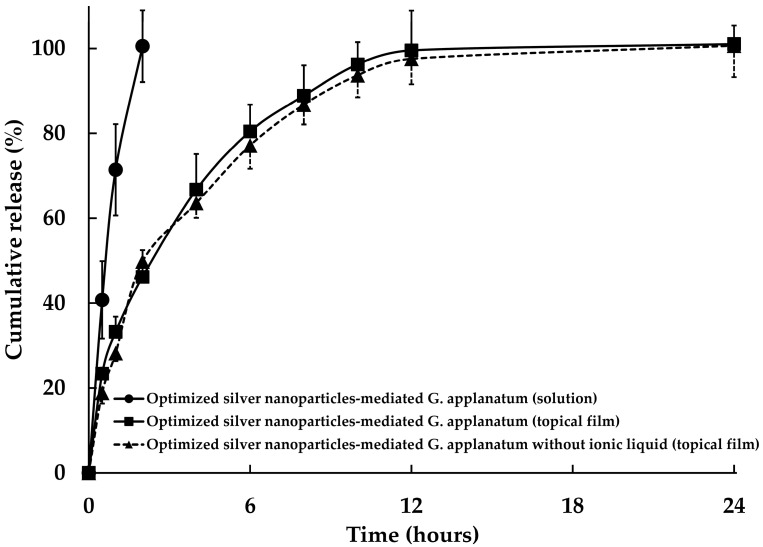
Release profile of silver-nanoparticle-complexed *G. applanatum* from the topical film.

Figure 14 shows the release profiles of the silver-nanoparticle-complexed *G. applanatum* from the solution and topical film. The silver-nanoparticle-complexed *G. applanatum* was able to quickly diffuse and be released from the solution into the receptor medium within 2 h. This occurred because the solution was easily dispersed in water and rapidly passed through the cellulose dialysis membrane. Furthermore, the silver-nanoparticle-complexed *G. applanatum* was easily released from the topical film. When the receptor medium entered the polymer matrix, there was a chance that the matrix would have swelled or eroded, which would then have caused the diffusion of the active compound or its dissolution [38]. For the silver-nanoparticle-complexed *G. applanatum* without ionic liquid, distilled water was used in place of the ionic liquid during the preparation procedure; it was subsequently mixed with the topical film. It was found that its release profile from the topical film did not show significant differences from the original silver-nanoparticle-complexed *G. applanatum*.

#### 3.2.4. Kinetic Models

The release of silver-nanoparticle-complexed *G. applanatum* from their formula was evident, as shown by a comparison of the R^2^ values of the zero-order, first-order, and Higuchi models (as shown in Table 4), wherein the R^2^ value of the Higuchi model was higher in both cases than the values of the zero-order and first-order models. Additionally, it was clear that the release of the silver-nanoparticle-complexed *G. applanatum* was controlled by both diffusion and dissolution mechanisms, which suggests that the drug was released from the topical film [39]. The Higuchi model, as the most precise model, was used to estimate the release rates of the silver-nanoparticle-complexed *G. applanatum* from the solution and the topical film. The release rate of the silver-nanoparticle-complexed *G. applanatum* from the solution was higher than its release rates from the topical films, which were correlated with the release profile (Figure 14). These findings were due to the fact that the silver-nanoparticle-complexed *G. applanatum* was able to diffuse and be released from the solution into the receptor media within 2 h. The release rate of the silver-nanoparticle-complexed *G. applanatum* from the topical film was decreased from 30.942 ± 2.444 %/$\sqrt{\min}$ to 30.060 ± 2.757 %/$\sqrt{\min}$ when the ionic liquid was replaced with distilled water. Thus, there is no effect when replacing the ionic liquid with distilled water.

The release of the silver-nanoparticle-complexed *G. applanatum* from the topical films showed diffusion exponent *n*-values of 0.440 and 0.478, according to the Korsmeyer–Peppas model. Because the diffusion exponent *n*-values were less than 0.5, Fickian diffusion was utilized to describe how the silver-nanoparticle-complexed *G. applanatum* was released from the topical films. When the polymer relaxation time is significantly longer than the typical solvent diffusion period, the process of solute transport is referred to as Fickian diffusion [38,40,41]. In conclusion, in this study, it was shown that the release of the silver-nanoparticle-complexed *G. applanatum* from the topical films was most probably caused by a diffusion-controlled process.

#### 3.2.5. In Vitro Permeation of Silver-Nanoparticle-Complexed *G. applanatum* from Topical Film

**Figure 15:**
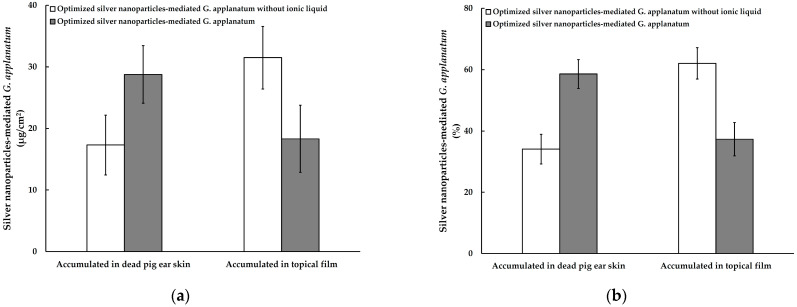
Silver-nanoparticle-complexed *G. applanatum* accumulation in dead pig ear skin and topical film: (**a**) content and (**b**) percentage of silver-nanoparticle-complexed *G. applanatum*.

An in vitro permeation investigation was carried out to evaluate the potential for silver-nanoparticle-complexed *G. applanatum* dispersions to permeate the dermis and epidermis of dead pig ear skin. The permeation of potentially topical drugs is often assessed using in vitro methods involving skin taken from an animal. The stratum corneum’s permeability characteristics, which may be thought of as the rate-limiting stage for skin absorption, remain unaltered after its removal from the body, allowing for direct comparison [42]. In this study, less than 5% of the silver-nanoparticle-complexed *G. applanatum*’s in vitro skin permeation through the dead pig ear skin into the receptor medium was detected after 24 h of testing, suggesting there was a low amount of silver-nanoparticle-complexed *G. applanatum* permeation. The stratum corneum acted as a barrier to the silver-nanoparticle-complexed *G. applanatum*’s permeation through the dead pig ear skin, in contrast to the in vitro release investigation [38,43]. These findings are consistent with other studies, which found that when skin is assessed with intact tissue, fewer silver nanoparticles are found in the receptor compartment [42,44]. Figure 15 shows the accumulation of the silver-nanoparticle-complexed *G. applanatum* following complete in vitro skin permeation. The quantity of silver-nanoparticle-complexed *G. applanatum* that accumulated in the dead pig ear skin after the 24-h in vitro permeation study was 28.77 ± 4.68 μg/cm^2^ (58.61 ± 9.53 %), while the quantity of the silver-nanoparticle-complexed *G. applanatum* without ionic liquid that accumulated was 17.30 ± 4.86 μg/cm^2^ (34.09 ± 9.57 %). Meanwhile, the amount of silver-nanoparticle-complexed *G. applanatum* deposition in the topical film was lower than that of the silver-nanoparticle-complexed *G. applanatum* without ionic liquid. Thus, it was evident from the diffusion investigation that for the small-sized silver-nanoparticle-complexed *G. applanatum*, more nanoparticles retained localized in the skin, and fewer of these penetrated the receptor compartment. According to studies on silver [42] and metallic [45] nanoparticles, small nanoparticles may penetrate the skin’s stratum corneum and hair follicle orifices and reach the stratum corneum’s deepest levels while remaining outside the skin’s surface. These studies hypothesized that because these nanoparticles have such a localizing impact on the skin, they will be useful in topical applications that reduce toxic effects.

In addition to their traditional uses as synthesis catalysts and solvents in chemistry, ionic drugs have been used in pharmaceutical applications in order to improve the dissolution, solubility, and bioavailability of drugs. When making an ionic liquid, inactive counter ions are typically used [46,47]. In the current investigation, an ionic liquid based on 1-butyl-3-methylimidazolium bromide appeared to demonstrate some efficacy in increasing silver-nanoparticle-complexed *G. applanatum* skin permeability, namely by approximately 1.7 fold. The cause of this improvement was the lipid extraction of the ionic liquid based on 1-butyl-3-methylimidazolium bromide, which might also have diffused into the stratum corneum, reduced the inter-lipid interactions, and extracted the lipids into the ionic liquid reservoir on the skin surface, thereby increasing the silver-nanoparticle-complexed *G. applanatum* permeation into the skin. Similar to this research, ionic liquids have recently been used to solve the permeability and solubility issues that the majority of drugs designed for skin delivery experience [48,49].

## 4. Conclusions

The purpose of this work was to develop and investigate the effect of ionic liquid based on 1-butyl-3-methylimidazolium bromide on the silver-nanoparticle-complexed *G. applanatum* loaded into a topical film. The experiments carried out employed the best ratio and preparation conditions. The optimal ratio of silver nanoparticles: *G. applanatum* extract: ionic liquid was set at 97:1:2, with the mixture incubating for an hour at 80 °C. As the prediction had a low percentage error, it was verified. The silver-nanoparticle-complexed *G. applanatum* solution was transparent and dark brown. The maximum peak absorption of the silver-nanoparticle-complexed *G. applanatum* was detected in the UV–visible absorption spectra at 405.5 nm. The optimized silver-nanoparticle-complexed *G. applanatum* was loaded into a topical film made of PVA and Eudragit^®^, and its properties were evaluated. When the silver-nanoparticle-complexed *G. applanatum* was loaded into the topical film, the film became yellow. The topical film had many desired properties, as well as being uniform, smooth, and compact. Furthermore, silver-nanoparticle-complexed *G. applanatum* was detected in the topical film. The topical film’s components were all compatible; therefore, even if there were small changes to any of them, they had no effect. It was calculated that there was 736.31 ± 138.82 µg of silver-nanoparticle-complexed *G. applanatum* in the topical film, which had a surface area of 15 cm^2^. This product may be utilized for effective applications. Furthermore, the concentration in the topical film was more potent than the IC_50_ estimation. The topical film also had control over the matrix-layer-mediated release of the silver-nanoparticle-complexed *G. applanatum*. The release kinetic was fitted using the Higuchi model. It was demonstrated that a diffusion-controlled process was most likely to be responsible for the release of the silver-nanoparticle-complexed *G. applanatum* from the topical films. In a 24-h in vitro permeation investigation, the quantity of the silver-nanoparticle-complexed *G. applanatum* that accumulated in the dead pig ear skin was 28.77 ± 4.68 μg/cm^2^ (58.61 ± 9.53 %), whereas the quantity of the silver-nanoparticle-complexed *G. applanatum* without ionic liquid that accumulated was 17.30 ± 4.86 μg/cm^2^ (34.09 ± 9.57 %). Thus, ionic liquid based on 1-butyl-3-methylimidazolium bromide, which might improve solubility, increased the skin accumulation of the silver-nanoparticle-complexed *G. applanatum* by about 1.7 times. Furthermore, it was clear from the research that the silver-nanoparticle-complexed *G. applanatum* remained in the skin rather than penetrating the receptor compartment. The produced film is appropriate for topical applications and may be used in the development of potential future pharmaceutical products for the treatment of diseases.

## Figures and Tables

**Table 1:** Ratio optimization of silver nanoparticles (*X*_1_), *G. applanatum* extract (*X*_2_), and ionic liquid (*X*_3_) by the Design-Expert^®^ program (Stat-Ease, Inc.).

Sample	Silver Nanoparticles	*G. applanatum* Extract	Ionic Liquid
	(*X*_1_)	(*X*_2_)	(*X*_3_)
1.1	91	1	4
1.2	97	1	4
1.3	91	3	4
1.4	97	3	4
1.5	91	2	2
1.6	97	2	2
1.7	91	2	6
1.8	97	2	6
1.9	94	1	2
1.10	94	3	2
1.11	94	1	6
1.12	94	3	6
1.13	94	2	4

**Table 2:** Condition optimization for ratio optimization shown in Table 1 by the Design-Expert^®^ program (Stat-Ease, Inc.).

Sample	Temperature (°C)	Time (h)
	(*X*_4_)	(*X*_5_)
2.1	40	1
2.2	40	2
2.3	40	3
2.4	60	1
2.5	60	2
2.6	60	3
2.7	80	1
2.8	80	2
2.9	80	3

**Table 3:** Identification of the main peaks from reported FTIR spectra.

Wavenumber (cm^−1^)	Peak Assignment
Silver-nanoparticle-complexed *G. applanatum*	
3444	O–H stretching in alcohol and phenol and N–H stretching in primary and secondary amide
2923	C–H stretching
1642	C=N stretching in amide and C=O stretching in an unsaturated aromatic carboxylic acid
1457	C=O and N–O stretching in ester and nitro groups
1377	C–O stretching in an aromatic compound
1024	C–F stretching in fluroalkanes
936	C=C stretching in alkanes and O–H stretching
Blank film	
3309	O–H stretching
2938, 2892	C–H stretching
1730	C=O stretching in ester
1443	C–H bending
1241	C–O stretching or –O–CH_2_–C
1110	C–C stretching and C–H bending
1036	C–O stretching and C–H bending
848	O–H bending

**Table 4:** Kinetic models of release for the silver-nanoparticle-complexed *G. applanatum* profiles from their formula.

	R^2^			*n*	Release Rate (K_H_) * (%/$\sqrt{\min}$)
	Zero Order	First Order	Higuchi	Korsmeyer–Peppas	
Silver-nanoparticle-complexed *G. applanatum* from the solution	0.9651	0.9899	0.9909	-	69.254 ± 7.566
Silver-nanoparticle-complexed *G. applanatum* from the topical film	0.9379	0.9923	0.9932	0.440	30.942 ± 2.444
Silver-nanoparticle-complexed *G. applanatum* from the topical film (without ionic liquid)	0.9326	0.9876	0.9886	0.478	30.060 ± 2.757

* Calculated from the Higuchi model.

## Data Availability

Not applicable.
